# Neoadjuvant Multiagent Systemic Therapy Approach to Liver Transplantation for Perihilar Cholangiocarcinoma

**DOI:** 10.1097/TXD.0000000000001760

**Published:** 2025-02-07

**Authors:** Nadine Soliman, Ashton A. Connor, Ashish Saharia, Sudha Kodali, Ahmed Elaileh, Khush Patel, Samar Semaan, Tamneet Basra, David W. Victor, Caroline J. Simon, Yee Lee Cheah, Mark J. Hobeika, Constance M. Mobley, Mukul Divatia, Sadhna Dhingra, Mary Schwartz, Anaum Maqsood, Kirk Heyne, Maen Abdelrahim, Milind Javle, Jean-Nicolas Vauthey, A. Osama Gaber, R. Mark Ghobrial

**Affiliations:** 1 Faculty of Biology, Medicine, and Health, University of Manchester, Manchester, United Kingdom.; 2 Department of Surgery, Houston Methodist Hospital, Houston, TX.; 3 Department of Surgery, Weill Cornell Medical College, New York, NY.; 4 Sherrie and Alan Conover Center for Liver Disease and Transplantation, Department of Medicine, Houston Methodist Hospital, Houston, TX.; 5 Department of Medicine, Weill Cornell Medical College, New York, NY.; 6 Department of Pathology and Genomic Medicine, Houston Methodist Hospital, Houston, TX.; 7 Division of Medical Oncology, Department of Medicine, Houston Methodist Hospital, Houston, TX.; 8 Department of Medical Oncology, University of Texas MD Anderson Cancer Center, Houston, TX.; 9 Department of Surgical Oncology, University of Texas MD Anderson Cancer Center, Houston, TX.

## Abstract

**Background.:**

Perihilar cholangiocarcinoma (phCCA) has excellent outcomes following liver transplantation (LT). Neoadjuvant radiation-based locoregional therapy is standard-of-care. Gemcitabine and cisplatin (gem/cis) combination systemic therapies have improved outcomes in advanced settings, but their efficacy pre-LT has not been studied.

**Methods.:**

We review our experience following neoadjuvant gem/cis alone versus radiation-based approaches. Patients with phCCA undergoing LT at a single center between January 2008 and February 2023 were identified retrospectively. Neoadjuvant therapy was categorized as gem/cis systemic therapy (ST) alone, or any ST and radiotherapy (RT). Outcomes were posttransplant overall survival (OS), recurrence-free survival (RFS), waitlist time, and pathologic tumor response.

**Results.:**

During study period, 27 phCCA patients underwent LT. One patient decompensated with neoadjuvant therapy and was excluded. Median age was 61 y (interquartile range, 53–68 y) and 14 (54%) were male. Of 26 patients, 12 (46%) received ST and 14 (54%) RT. Six RT patients received gem/cis ST. Median waitlist time was 199 d (interquartile range, 98–405 d) and did not differ by neoadjuvant regimen. Explanted tumors were predominantly T1 stage, without lymphovascular invasion or nodal involvement. Neither pathologic features nor percent tumor necrosis differed by regimen. OS probabilities at 1 and 3 y were 84% and 55% for the cohort. There was no significant difference in OS and RFS when stratified by regimen.

**Conclusions.:**

Post-LT OS, RFS, waitlist time, and tumor response were similar in the 2 groups. Patients with phCCA who do not undergo RT may still be considered for LT under appropriate institution-based protocols that adhere to other established criteria.

Perihilar cholangiocarcinoma (phCCA) has increasing incidence and mortality rates in the United States and globally. In 2020, >90 000 new cases and 83 000 deaths were reported.^1^ Patients who present with localized disease involving only the common bile duct, first-order bile ducts or even unilateral second-order ducts and undergo resection have better prognosis, with reported 5-y overall survival (OS) of 30%–65%,^2,3^ whereas those with locally advanced disease who are anatomically unresectable have dismal 5-y OS of <2%.^4,5^

Since 2000, liver transplantation (LT) has emerged as a viable treatment option for patients with locally advanced phCCA, with reported 5-y OS also ranging from 30% to 65%.^6-8^ Outcomes are dependent on use of neoadjuvant therapy, final tumor pathology (including residual tumor, lymphovascular invasion [LVI], and nodal metastases), underlying primary sclerosing cholangitis (PSC), and center experience.^8-11^ The most common neoadjuvant regimen involves external beam radiation therapy (RT) of 40–55 Gy given in 30 fractions >3–5 wk alongside continuous 5-fluorouracil (5FU) infusion, followed by intraductal brachytherapy of 8–60 Gy iridium-192 with concomitant 5FU or capecitabine, which is continued until time of transplant.^12^ Deviations have been reported but all include RT. Dropout rates range from 10% to 67%.^8,13^ This neoadjuvant protocol was developed before the establishment of effective systemic therapy (ST) for phCCA in the palliative setting. In 2010, gemcitabine and cisplatin (gem/cis) combination were shown to have a superior response rate, progression-free and OS compared with gem monotherapy, with no increase in toxicity.^14^ Subsequent studies have shown benefit of adding immunotherapy to this combination,^15,16^ the safety of which has not been confirmed before transplantation.^17^ Use of these combination regimens in the neoadjuvant setting before LT without RT have not been assessed. Following advances in ST for other cancers, including pancreatic and rectal carcinomas, benefit of RT in the neoadjuvant setting has been called into question.^18-20^

Here, we describe our centers’ experience with LT for phCCA to evaluate differences in neoadjuvant therapy utilizing either ST alone or combined ST and RT. Outcomes include waitlist time, degree of response on explant pathology, recurrence-free survival (RFS), and OS post-LT. We aim to show that LT for locally advanced phCCA patients who do not undergo RT is a viable option with modern systemic therapies.

## MATERIALS AND METHODS

### Study Subjects

Patients were identified retrospectively from a clinical database. Inclusion criteria were histologic diagnosis of phCCA and LT between January 2008 and February 2023. Variables extracted included underlying etiologies such as PSC, explanted tumor pathology, treatment types and dates, recurrence rates and last follow-up. The study protocol was approved by the Houston Methodist Research Institutional Review Board with a waiver of informed consent (PRO00000587). Our transplant center adheres to United Network for Organ Sharing requirements for phCCA Model for End-Stage Liver Disease score exceptions and to Organ Procurement and Transplantation Network clinical practice guidelines for pre-LT management. This includes a written protocol to the Liver and Intestinal Organ Transplantation Committee outlining selection criteria, neoadjuvant therapy, and operative staging. All patients had pre-LT endoscopic biopsy or cytology evidence of cholangiocarcinoma (CCA), were deemed unresectable by multidisciplinary cancer conference consensus, had cross-sectional imaging demonstrating a single lesion <3 cm in maximum size without extrahepatic spread, and had no lymph node or peritoneal involvement on operative staging after completion of neoadjuvant therapy.

### Exposures

Neoadjuvant treatment regimens were prescribed at the discretion of the multidisciplinary clinical team and not made in a prospective, centralized fashion. Frequently, decisions for first-line therapy are made by teams at the referring institution alone. For analyses, treatments were stratified as either pretreatment including RT or not. Candidacy for LT was determined in standard multidisciplinary fashion at our institution.

### Outcomes

Primary outcome was OS from the date of LT. Secondary outcomes included time from waitlisting to transplantation, degree of tumor response to neoadjuvant therapy on explanted liver pathology, and RFS. Treatment response on explant pathology was quantified by degree of tumor necrosis, both as a continuous variable and stratified as complete (100% necrosis), incomplete (1%–99%), or none (0%).

### Statistical Analysis

Association of clinicopathologic variables with time-to-event outcomes, including OS, RFS, and waitlist time, were determined by Cox proportional hazards models. For exposures occurring a fixed time after LT (ie, use of adjuvant therapy), the landmark method was used. Associations of continuous and categorical variables with cohort strata were determined by Kruskal-Wallis and Fisher exact tests, respectively. All analyses were performed in R version 4.3.2.

## RESULTS

### Cohort Features

**FIGURE 1.**
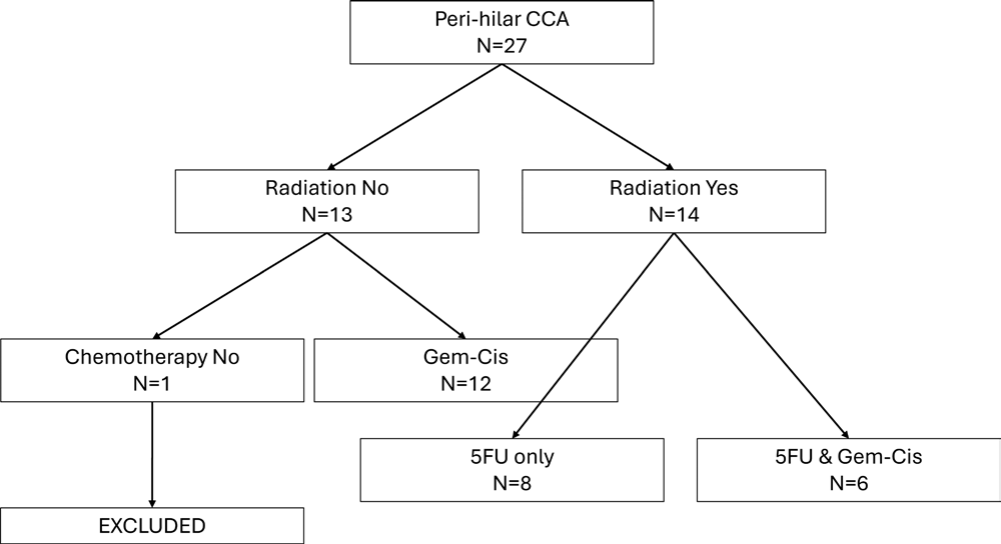
Flow diagram of study design. 5FU, 5-fluorouracil; CCA, cholangiocarcinoma; Gem-Cis, gemcitabine and cisplatin.

Our cohort included 27 patients who underwent LT for phCCA between January 2008 and February 2023 (Figure 1). Of the 27 patients, 26 received neoadjuvant therapy. One (1) patient transplanted for decompensated cirrhosis, did not receive any neoadjuvant regimen, and was therefore excluded from downstream analyses. Of the remaining 26 patients, 14 were given single agent 5-FU and RT, 6 of whom also received combination gem/cis ST, and 12 had combination gem/cis ST for at least 6 cycles alone without RT, preoperatively (Figure 1). RT regimens included external beam RT in 13 patients, brachytherapy in 3, and stereotactic body radiotherapy in 1 (Table 1). All completed their planned radiation. The duration of gem/cis did not significantly differ in the 2 cohorts. Patients who received gem/cis were maintained on twice weekly gem until transplant. Patients who received 5-FU and radiation alone were maintained on either 5-FU or oral capecitabine until transplant. Regimens were not significantly associated with the year of LT (*P* = 0.051).

**TABLE 1.** Baseline recipient features stratified by neoadjuvant treatment

Variable	Total (N = 26)	Neoadjuvant treatment regimen		*P*
		No radiation (N = 12)	Radiation (N = 14)	
Age at transplant, median (IQR)	61.0 (53.0–68.0)	60.5 (58.5–63.5)	61.5 (47.0–71.0)	1.00
Female sex, n (%)	12 (46.2)	2 (16.7)	10 (71.4)	**0.02**
Diagnosis of PSC/PBC, n (%)	6 (23.1)	2 (16.7)	4 (28.6)	0.77
MELD at transplant, median (IQR)	9.0 (8.0–19.0)	8.0 (7.0–10.0)	13.0 (8.0–21.0)	0.12
Maximum pretransplant CA19-9, U/mL, median (IQR)^*a*^	81.5 (33.0–339.0)	43.0 (20.5–234.5)	118.0 (63.0–301.5)	0.30
Time on waitlist, d, median (IQR)	199.0 (98.0–405.0)	223.5 (93.0–408.0)	180.5 (98.0–405.0)	0.67
Pretransplant hepatectomy, n (%)	1 (3.8)	0 (0)	1 (7.1)	1.00
Peritransplant Whipple, n (%)	5 (19.2)	1 (8.3)	4 (28.6)	0.42
Neoadjuvant gemcitabine and cisplatin, n (%)	18 (69.2)	12 (100)	6 (42.9)	**0.007**
Neoadjuvant gemcitabine and cisplatin duration, d, median (IQR)	126.0 (107.0–186.8)	138.0 (109.0–190.5)	117.5 (81.0–165.2)	0.94
External beam radiation therapy, n (%)	13 (50)	0 (0)	13 (92.9)	
Stereotactic body radiation therapy, n (%)	1 (3.8)	0 (0)	1 (7.1)	
Brachytherapy, n (%)	3 (11.5)	0 (0)	3 (21.4)	
Radiation dose (Gy), median (IQR)			60 (52.5–70)	

Bold values denote *P* < 0.05.

^*a*^Available for 22 patients.

CA19-9, carbohydrate antigen 19-9; IQR, interquartile range; MELD, Model for End-Stage Liver Disease; PBC, primary biliary cirrhosis; PSC, primary sclerosing cholangitis.

### Baseline Patient Features

Baseline patient variables were well balanced between the 2 neoadjuvant strata (Table 1; **Table S1, SDC**, http://links.lww.com/TXD/A737). Six (6) patients had a predisposing autoimmune condition, either PSC or primary biliary cirrhosis, while 18 tumors were sporadic. The median age at LT was 61 (interquartile range [IQR], 53–68). The cohort consisted of 12 (46.2%) female and 14 (52.8%) male patients. Interestingly, more female (10) than male patients (4) received RT in the neoadjuvant setting (*P* = 0.016). Median biological Model for End-Stage Liver Disease score pretransplant was 9 (IQR, 8–19). Only 1 patient underwent pretransplant hepatectomy. Carbohydrate antigen 19-9 values did not significantly differ at any time point between groups.

### Waitlist Times and Donor Features

**FIGURE 2.**
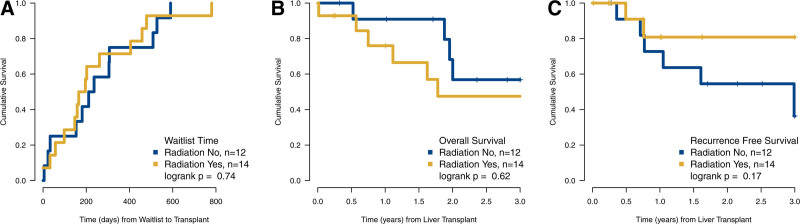
Time to event analysis. A, Cumulative incidence curve of time from waitlist to transplant. B, Overall patient survival from date of transplant to last follow-up or death. C, Recurrence-free survival from date of transplant to last follow-up or recurrence, for 26 perihilar cholangiocarcinoma patients stratified by neoadjuvant treatment regimens.

Median waitlist time was 199 d (IQR, 98–405 d) for the entire cohort (Table 1). This did not differ significantly across the 2 neoadjuvant strata (Figure 2A; *P* = 0.84). Donor features were balanced across the 2 strata (**Table S2, SDC**, http://links.lww.com/TXD/A737). All were deceased donors, including 23 brain deaths and 3 circulatory deaths. Median kidney donor profile index was 37.5 (IQR, 18–82), and median donor age was 41.5 (IQR, 25–52). Median distance from donor to recipient hospital was 205.5 miles (IQR, 127–267 miles), with 13 (50%) donors found locally, 8 (30.8%) regionally, and 5 (19.2%) nationally. Donor features were not associated with outcomes.

### Operative Features

Operative features were also well balanced across the 2 groups (Table 1). A minority of patients (n = 5/26) in each neoadjuvant strata required Whipple procedures to achieve negative margins, implying no differences in locally advanced cases. Median cold ischemia time was 5.5 h (IQR, 4.9–8.3 h) and did not significantly differ between strata. Estimated blood loss and volumes of blood products transfused trended toward greater values in the strata receiving RT (**Table S1, SDC**, http://links.lww.com/TXD/A737).

### Explant Pathology and Biomarker Features

As per study inclusion criteria, all patients had histologic evidence of invasive phCCA on either pretransplant biopsy or explanted liver pathology. In the latter (Table 2), 18 (69.2%) patients had residual adenocarcinoma histologically. Median tumor size was 2.5 cm (IQR, 1.2–3.5 cm), and most tumors were solitary. Most were moderately differentiated without LVI. Six (6) cases had nodal metastases. Histology did not differ across the 2 strata. Treatment effect as percent tumor necrosis was a median 67.5% (IQR, 0%–100%) and did not differ between the 2 treatment types (*P* = 0.54; Table 2). When stratified as complete (n = 8, 33.3%), incomplete (n = 9, 37.5%), and none (n = 7, 29.2%), treatment effect also did not significantly differ (*P* = 0.69). There was a nonsignificant trend toward less treatment response in patients sequentially receiving both RT/5FU and gem/cis therapy preoperatively, with no complete responders, implying that these were more recalcitrant tumors requiring more therapy.

**TABLE 2.** Explanted liver pathology features stratified by recipient neoadjuvant treatment

Variable	N	Total (N = 26)	Neoadjuvant treatment regimen		*P*
			No radiation (N = 12)	Radiation (N = 14)	
T stage, n (%)	26				
T0/Tis		8 (30.8)	5 (41.7)	3 (21.4)	0.327
T1		5 (19.2)	1 (8.3)	4 (28.6)	
T2/T3/T4		13 (50.0)	6 (50)	7 (50)	
N stage, n (%)					0.65
N0/Nx	26	20 (76.9)	10 (83.3)	10 (71.4)	
N1/N2		6 (23.1)	2 (16.7)	4 (28.6)	
Tumor grade/differentiation, n (%)	18				0.56
G1		3 (16.7)	1 (14.3)	2 (18.2)	
G2		12 (66.7)	4 (57.1)	8 (72.7)	
G3		3 (16.7)	2 (28.6)	1 (9.1)	
Focality, n (%)					1.00
Multifocal	26	3 (11.5)	1 (8.3)	2 (14.3)	
Solitary		23 (88.5)	11 (91.7)	12 (85.7)	
Maximum tumor size (cm), median (IQR)	22	2.5 (1.2–3.5)	3.0 (0.0–4.0)	2.3 (1.3–3.5)	1.00
Lymphovascular invasion, n (%)	26	5 (19.2)	2 (16.7)	3 (21.4)	1.00
Perineural invasion, n (%)	26	14 (53.8)	7 (58.3)	7 (50.0)	0.98
% tumor necrosis, median (IQR)	18	67.5 (0.0–100.0)	100.0 (0.0–100.0)	40.0 (0.0–100.0)	0.54
Treatment effect, n (%)					0.69
Complete	24	8 (33.3)	5 (41.7)	3 (25.0)	
Incomplete		9 (37.5)	4 (33.3)	5 (41.7)	
None		7 (29.2)	3 (25.0)	4 (33.3)	
Background liver cirrhosis, n (%)	26	10 (38.5)	4 (33.3)	6 (42.9)	0.93

IQR, interquartile range; Tis, tumor *in situ*.

### Recipient Posttransplant Outcomes

Median posttransplant follow-up was 668 d (IQR, 273–1163 d). In that period, there were 12 (46.2%) patient deaths (Table 3). For the entire cohort, probability of OS at 1 y was 83.0% (95% confidence interval [CI], 69.2%-99.7%) and at 3 y was 52.3% (95% CI, 34.5%-79.4%). On univariable analysis, LVI and nodal metastases were associated with OS and not neoadjuvant treatment regimen (Table 4; Figure 2B). While not statistically significant, the causes of death were always noncancer-related in the strata receiving RT, and predominantly cancer related in the strata that received only gem/cis (Table 3).

**TABLE 3.** Posttransplant outcomes stratified by recipient neoadjuvant treatment

Variable	Total (N = 26)	Neoadjuvant treatment regimen		*P*
		No radiation (N = 12)	Radiation (N = 14)	
Adjuvant systemic chemotherapy, n (%)	17 (65.4)	8 (66.7)	9 (64.3)	1.00
Recurrence, n (%)	9 (34.6)	6 (50)	3 (21.4)	
Recurrence-free survival, d, median (IQR)	485.0 (259. 0–1092.0)	605.5 (270. 0–1005.5)	371.5 (180. 0–1660.0)	0.17
Recurrence region, n (%)				
Local	6 (23.1)	4 (33.3)	2 (14.3)	0.50
Regional	4 (15.4)	3 (25)	1 (7.1)	0.48
Distal	4 (15.4)	3 (25)	1 (7.1)	0.48
Patient status at last follow-up, n (%)				
Alive	14 (53.8)	7 (58.3)	7 (50)	
Deceased	12 (46.2)	5 (41.7)	7 (50)	
Overall survival, d, median (IQR)	668.0 (273.0–1163.0)	722.5 (498.0–1064.5)	500.0 (208.0–1800.0)	0.62
Cause of death, n (%)				0.11
Cancer	7 (58.3)	5 (100)	2 (28.6)	
Cardiac	2 (16.7)	0 (0)	2 (28.6)	
Infection	1 (8.3)	0 (0)	1 (14.3)	
Multisystem organ failure	2 (16.7)	0 (0)	2 (28.6)	

IQR, interquartile range.

**TABLE 4.** Univariable Cox proportional hazards models for overall and recurrence-free survival postliver transplant

Variable	Overall survival		Recurrence-free survival	
	HR (univariable)	HR (multivariable)	HR (univariable)	HR (multivariable)
Pretransplant radiation therapy				
No	(Reference)		(Reference)	(Reference)
Yes	1.34 (0.41-4.36; *P* = 0.62)		0.34 (0.07-1.68; *P* = 0.19)	0.26 (0.05-1.31; *P* = 0.10)
Lymphovascular invasion				
No	(Reference)	(Reference)	(Reference)	
Yes	6.23 (1.75-22.09; *P* = 0.005)	10.12 (2.43-42.18; *P* = 0.001)	3.63 (0.81-16.32; *P* = 0.09)	
Maximum tumor size				
<3 cm	(Reference)		(Reference)	
>3 cm	2.88 (0.89-9.33; *P* = 0.08)		2.21 (0.59-8.31; *P* = 0.24)	
Nodal stage				
NX–N0	(Reference)		(Reference)	(Reference)
N1–N2	3.99 (1.20-13.25; *P* = 0.02)		5.06 (1.11-23.06; *P* = 0.04)	6.34 (1.38-29.21; *P* = 0.02)
Primary sclerosing cholangitis				
No	(Reference)		(Reference)	
Yes	0.96 (0.26-3.57; *P* = 0.95)		0.33 (0.04-2.67; *P* = 0.30)	
Age at transplant, y	0.95 (0.90-1.01; *P* = 0.11)		1.00 (0.93-1.08; *P* = 0.98)	
MELD score at transplant	1.07 (0.99-1.15; *P* = 0.10)	1.11 (1.01-1.22; *P* = 0.02)	0.97 (0.86-1.09; *P* = 0.59)	
Time on waitlist, d	1.00 (1.00-1.00; *P* = 0.88)		1.00 (1.00-1.01; *P* = 0.38)	
Liver cirrhosis				
No	(Reference)		(Reference)	
Yes	1.10 (0.35-3.52; *P* = 0.87)		1.07 (0.25-4.50; *P* = 0.93)	

HR, hazard ratio; MELD, Model for End-Stage Liver Disease.

There were 9 (34.6%) patients with cancer recurrence. Probability of RFS at 1 y was 77.8% (95% CI, 62.4%-97.0%) and at 3 y was 59.0% (95% CI, 39.7%-87.7%). The absolute number of recurrences in the 2 strata was not significantly different (Table 3). By log-rank test, the times to recurrence were nonsignificant between the 2 strata (*P* = 0.71; Figure 2C). Only nodal metastases were associated with worse RFS by Cox analysis (Table 4). Also, sites of first recurrence did not differ, with most recurrences being local (6, 23%), within the hepatic allograft.

There were 17 patients (65.4%) who received adjuvant ST consisting of oral capecitabine starting approximately 30 d from the date of transplant for an intended 8 cycles (Table 3).^21,22^ On univariable Cox proportional hazard models adjusting for 30 d by landmark method, OS was again associated with LVI and nodal status, as well as age at transplant, and RFS was only associated with nodal status (**Table S3, SDC**, http://links.lww.com/TXD/A737; **Figure S1A** and **B, SDC**, http://links.lww.com/TXD/A737). There were no recurrences among patients who did not receive capecitabine (*P* = 0.0088).

## DISCUSSION

In this retrospective cohort study of LT for unresectable phCCA, administration of neoadjuvant RT had no statistically significant association with either pre-LT waitlist time, post-LT OS or treatment effect on explanted liver pathology. There was a possible association of more blood loss, more blood product transfusion, and shorter RFS with use of RT. Patients with LVI and nodal metastases had worse OS, and the latter also had worse RFS. Adjuvant capecitabine was not associated with outcomes but was not used universally, as many patients were treated before publication of the capecitabine compared with observation in resected biliary tract cancer (BILCAP) studies.^21,22^ This data suggests that neoadjuvant regimens using combination systemic therapies may safely substitute RT-based protocols.

Early outcomes for LT for locally advanced phCCA were disappointing, with retrospective series reporting high recurrence rates and short survival.^23^ Addition of RT-based neoadjuvant therapy improved outcomes considerably, with series reporting approximate 3-y recurrence rates of 25% and survival of 65%. Subsequent randomized controlled trials (RCTs) have investigated the use of combination ST in unresectable CCA with promising results. The Advaned Biliary tract Cancer (ABC)-01, -02, and -03 studies included 72 phCCA patients with median progression-free survival of 8.4 mo, median OS of 12.2 mo, and an objective radiological response rate of 18.4%.^24^ The TOPAZ-1 trial randomized 685 biliary tract cancer patients to gem/cis with or without durvalumab, with median OS of 12.9 and 11.3 mo, respectively.^25^ The KEYNOTE-966 trial randomized 1069 biliary tract cancer patients gem/cis with or without pembrolizumab, with median OS of 12.7 and 10.9 mo, respectively.^15^ These studies suggest that application of these multimodal systemic therapies to the pre-LT setting might offer good outcomes for phCCA patients without RT, as recently demonstrated with other cancers. In locally advanced rectal cancer, landmark randomized trials showed 10-y local recurrence of <10% with use of neoadjuvant RT-based therapy.^26,27^ However, recent randomized^20^ and cross-sectional^19^ evidence suggests that equivalent local recurrence rates of approximately 5% can be maintained with omission or more restrictive use of radiation in neoadjuvant protocols that include modern combination systemic therapies.^19,20^ Similarly in borderline resectable pancreatic cancer, a recent RCT showed superior postoperative survival in patients receiving neoadjuvant therapy, either systemic alone or with radiation, compared with immediate surgery.^28^ Another recent RCT by Katz et al^18^ compared neoadjuvant ST with or without RT in patients with borderline unresectable pancreatic ductal adenocarcinoma (PDAC), finding that addition of RT was not more effective than ST alone. Given these advancements in neoadjuvant therapy for other cancers, it is both timely to review the role of radiation in the neoadjuvant setting for phCCA and to expect that some patients can achieve equivalent outcomes with its omission.

There are potential benefits to ST-only over RT-based approaches. Response rates of CCA to RT reportedly range from 70% to 80% in dose-dependent manner.^29,30^ Objective tumor response rates of CCA to single agent ST with gem is only approximately 70% compared with 80% with combination gem/cis.^14^ RT-based protocols have high patient drop-out rates, ranging from 10% to 67%,^8,13^ although waitlist outcomes are under-reported.^8^ Additionally, a recent European study suggested use of neoadjuvant chemotherapy alone after noting a higher rate of hepatic vascular complications in patients receiving neoadjuvant chemoradiotherapy, citing supporting evidence from several notable studies.^31-34^ In a multicenter study, reported toxicities to perihilar RT include peptic ulcers, portal vein, hepatic artery stenosis and/or thrombosis, and biliary leak and strictures, in 10%–35% of transplanted patients.^10^ In rectal cancer, postoperative complication rates are greater and functional outcomes are worse in patients treated with RT-based neoadjuvant therapy compared with only systemic.^19^ More so, RT-based protocols require patient selection for LT to be made upfront, with inherently less fluidity than systemic-therapy based approaches. Allowance of combination systemic therapies neoadjuvantly may permit greater accessibility for patients initially treated with palliative intent to be reconsidered for LT and patients treated at centers without access to biliary RT.

The 1- and 3-y OS probabilities observed in this cohort were 83% and 52.3%. These agree with those recently reported in international meta-analysis and retrospective national American cohorts.^8,9^ The national American experience suggests a volume-outcome relationship for LT for phCCA, with higher volume centers achieving 3-y OS of 56.9% compared with 48.8% in lower volume centers, with a cutoff of 6 LTs for phCCA per center.^9^ Many series of LT for phCCA report only 1-y outcomes,^8^ demonstrating a need for longer term reports, such as in this study. This study found that worse outcomes were predominantly associated with tumor biology (LVI, nodal stage), suggesting that a personalized medicine approach with genomic markers for both prognosis and prediction of therapy response may play future roles in this space.^35-41^ There may be patients in whom it is oncologically safe to omit RT, and others who will predictably benefit from it. Certain pathologic features, such as tumor size, were not significant in our cohort, as our center abides by United Network for Organ Sharing guidelines for phCCA transplantation, which include radiological size limits.^4,13^

The primary weaknesses of this study were its small sample size and real-world, retrospective, nonrandomized structure. Treatment decisions were not made in a centralized, prospective fashion. Thus, there is a possible selection bias that prohibits comment on the superiority of either treatment arm. Our sample size, however, is larger than that of most single center studies on LT for phCCA.^8^ More so, there are no published randomized control trials for phCCA and LT, although at least 1 is open.^42^ Additionally, our study only includes patients who underwent LT, so we cannot comment on dropout in either neoadjuvant treatment arm, a weakness of most series on LT for phCCA.^8^ Also, there was a possible selection bias of females receiving pre-LT RT. A primary strength of this study is the high granularity of patient information. To our knowledge, this is the first study to report on combination ST in the neoadjuvant setting for LT for phCCA. There is a need for future prospective studies to validate the hypothesis generated here that excellent post-LT outcomes can be obtained in some phCCA patients treated without neoadjuvant ST alone.

Overall, this series suggests that neoadjuvant combination systemic therapies alone may achieve equivalent LT outcomes to RT-based neoadjuvant therapies. This finding of possible therapeutic equivalence of neoadjuvant combination ST regimens with RT-based regimens mirrors that recently reported in neoadjuvant pancreatic and rectal cancer studies.^18,19^ This implies that greater access to LT may be permissible to patients with locally advanced phCCA, who otherwise lack treatment options and have dismal prognosis. This merits validation in external cohorts, and future efforts may integrate genomic markers of prognosis and targeted ST susceptibility.

## CONCLUSIONS

In this retrospective, single-center study, 26 patients underwent LT for phCCA and were stratified into 2 groups based on neoadjuvant treatment regimens. No significant differences in waitlist time, histological treatment response and OS were observed among the groups. This study implies that patients with locally advanced phCCA who do not undergo RT may still be considered for LT and expect excellent outcomes under appropriate institution-based protocols that adhere to other established criteria for transplant.

## Supplementary Material


