# Supplementary material for: Neoadjuvant Multiagent Systemic Therapy Approach to Liver Transplantation for Perihilar Cholangiocarcinoma
Source: Transplant Direct. 2025 Feb 7;11(3):e1760. doi: 10.1097/TXD.0000000000001760 (PMC11809964; doi:10.1097/TXD.0000000000001760)

Supplementary Material

Supplementary Table 1: Additional recipient features stratified by neoadjuvant treatment.

			Neoadjuvant Treatment Regimen		
**Variable**	**Strata**	**Total (N=26)**	**Radiation No (N=12)**	**Radiation Yes (N=14)**	**p**
**Baseline Recipient Features**					
BMI at transplant	Median (IQR)	26.7 (22.8 to 29.6)	27.5 (24.2 to 28.5)	24.6 (21.6 to 30.1)	0.569
Condition at transplant	Home	23 (88.5%)	11 (91.7%)	12 (85.7%)	0.639
	Hospital	1 (3.8%)	0 (0%)	1 (7.1%)	
	ICU	2 (7.7%)	1 (8.3%)	1 (7.1%)	
CA19-9 Level At Referral	Median (IQR)	94.0 (19.0 to 264.0)	25.0 (18.5 to 218.0)	117.0 (63.0 to 239.5)	0.412
CA19-9 Level At Evaluation	Median (IQR)	117.5 (19.0 to 273.0)	54.0 (19.0 to 406.5)	118.0 (63.0 to 239.5)	0.895
CA19-9 Level At Listing	Median (IQR)	117.5 (19.0 to 339.0)	41.0 (19.0 to 533.0)	118.0 (63.0 to 239.5)	0.895
CA19-9 Level At Transplant	Median (IQR)	59.0 (27.5 to 300.5)	42.0 (19.0 to 88.0)	112.5 (51.0 to 487.0)	0.143
Cold Ischemia Time (hours)	Median (IQR)	5.5 (4.9 to 8.3)	6.7 (5.0 to 8.7)	5.5 (4.4 to 8.0)	0.681
Volumes Transfused pRBCs (mL)	Median (IQR)	602.0 (300.0 to 1200.0)	300.0 (300.0 to 602.0)	900.0 (602.0 to 1204.0)	0.112
Volumes Transfused FFP (mL)	Median (IQR)	300.0 (150.0 to 583.0)	150.0 (150.0 to 150.0)	583.0 (301.0 to 600.0)	0.015
Volumes Transfused Albumin (mL)	Median (IQR)	1000.0 (750.0 to 1500.0)	1000.0 (1000.0 to 1500.0)	1000.0 (500.0 to 1500.0)	0.842
Estimated Blood Loss (mL)	Median (IQR)	1000.0 (1000.0 to 1500.0)	1000.0 (750.0 to 1000.0)	1500.0 (1000.0 to 2000.0)	0.12
Intra-Operative Urine Output (mL)	Median (IQR)	300.0 (200.0 to 600.0)	287.5 (200.0 to 600.0)	300.0 (175.0 to 700.0)	0.923
**Explant Pathology Features**					
T stage	T0	7 (26.9%)	5 (41.7%)	2 (14.3%)	0.237
	T1	5 (19.2%)	1 (8.3%)	4 (28.6%)	
	T2	11 (42.3%)	4 (33.3%)	7 (50%)	
	T3	1 (3.8%)	1 (8.3%)	0 (0%)	
	T4	1 (3.8%)	1 (8.3%)	0 (0%)	
	Tis	1 (3.8%)	0 (0%)	1 (7.1%)	
N stage	N0	16 (61.5%)	9 (75%)	7 (50%)	0.55
	N1	4 (15.4%)	1 (8.3%)	3 (21.4%)	
	N2	2 (7.7%)	1 (8.3%)	1 (7.1%)	
	Nx	4 (15.4%)	1 (8.3%)	3 (21.4%)	
M stage	M0	19 (73.1%)	10 (83.3%)	9 (64.3%)	0.455
	M1	1 (3.8%)	0 (0%)	1 (7.1%)	
	Mx	6 (23.1%)	2 (16.7%)	4 (28.6%)	
TNM stage	0	8 (30.8%)	5 (41.7%)	3 (21.4%)	0.461
	1	5 (19.2%)	1 (8.3%)	4 (28.6%)	
	2	9 (34.6%)	5 (41.7%)	4 (28.6%)	
	3	1 (3.8%)	0 (0%)	1 (7.1%)	
	4	3 (11.5%)	1 (8.3%)	2 (14.3%)	
**Post-Transplant Outcomes**					
Length of Hospital Stay (days)	Median (IQR)	15.0 (12.0 to 22.0)	12.0 (10.0 to 15.0)	17.0 (14.0 to 40.5)	**0.04**
Retransplantation	No	25 (96.2%)	11 (91.7%)	14 (100%)	0.937
	Yes	1 (3.8%)	1 (8.3%)	0 (0%)	
Post-Recurrence systemic therapy		6 (23.1%)	4 (33.3%)	2 (14.3%)	0.495
Site of Recurrence: Liver	No	20 (76.9%)	8 (66.7%)	12 (85.7%)	0.495
	Yes	6 (23.1%)	4 (33.3%)	2 (14.3%)	
Site of Recurrence: Lung	No	24 (92.3%)	11 (91.7%)	13 (92.9%)	1
	Yes	2 (7.7%)	1 (8.3%)	1 (7.1%)	
Site of Recurrence: Bone	No	23 (88.5%)	9 (75%)	14 (100%)	0.17
	Yes	3 (11.5%)	3 (25%)	0 (0%)	
Site of Recurrence: Peritoneum	No	23 (88.5%)	10 (83.3%)	13 (92.9%)	0.887
	Yes	3 (11.5%)	2 (16.7%)	1 (7.1%)	
Site of Recurrence: Portal LN	No	24 (92.3%)	10 (83.3%)	14 (100%)	0.394

Supplementary Table 2: Donor features stratified by recipient neoadjuvant treatment.

			Neoadjuvant Treatment Regimen		
**Variable**	**Strata**	**Total (N=26)**	**Radiation No (N=12)**	**Radiation Yes (N=14)**	**p**
Donor Type	DBD	23 (88.5%)	11 (91.7%)	12 (85.7%)	1
	DCD	3 (11.5%)	1 (8.3%)	2 (14.3%)	
KDPI continuous	Median (IQR)	37.5 (18.0 to 82.0)	37.5 (17.0 to 68.0)	37.0 (24.0 to 82.0)	0.837
KDPI stratified	(0,20]	7 (26.9%)	4 (33.3%)	3 (21.4%)	0.788
	(20,85]	14 (53.8%)	6 (50%)	8 (57.1%)	
	(85,100]	5 (19.2%)	2 (16.7%)	3 (21.4%)	
Donor age at procurement	Median (IQR)	41.5 (25.0 to 52.0)	38.5 (21.5 to 52.0)	45.0 (32.0 to 50.0)	0.471
Sex	female	10 (38.5%)	3 (25%)	7 (50%)	0.367
	male	16 (61.5%)	9 (75%)	7 (50%)	
Length of hospitalization pre-donation	Median (IQR)	4.0 (3.0 to 5.0)	4.0 (3.5 to 6.5)	4.0 (3.0 to 5.0)	0.319
Inter-hospital distance (km)	Median (IQR)	205.5 (127.0 to 267.0)	145.0 (15.0 to 237.0)	206.5 (202.0 to 382.0)	0.084
Share type	Local	13 (50%)	5 (41.7%)	8 (57.1%)	0.69
	Regional	8 (30.8%)	4 (33.3%)	4 (28.6%)	
	National	5 (19.2%)	3 (25%)	2 (14.3%)	

Supplementary Table 3: Univariable Cox proportional hazards models for overall and recurrence-free survival post-30-days from date of liver transplant (landmark method)

Variable	Strata	Overall Survival HR (univariable)	Recurrence Free Survival HR (univariable)
Adjuvant systemic		1.53 (0.33-7.11, p=.591)	
LVI	Present	8.22 (2.15-31.49, p=.002)	3.63 (0.81-16.32, p=.093)
Maximum tumor size	>3cm	3.68 (1.04-12.96, p=.043)	2.21 (0.59-8.31, p=.241)
N stage	N1-N2	5.02 (1.40-18.04, p=.014)	5.06 (1.11-23.06, p=.036)
Etiology	PSC/PBC	1.07 (0.28-4.05, p=.921)	0.33 (0.04-2.67, p=.297)
Recipient Age at Transplant (years)	Years	0.93 (0.87-1.00, p=.035)	1.00 (0.93-1.08, p=.977)
Recipient MELD at Transplant		1.06 (0.98-1.15, p=.169)	0.97 (0.86-1.09, p=.588)

Supplementary Figure 1: A. Overall and **B.** Recurrence-free survival from 30 days post-LT stratified by receipt of adjuvant chemotherapy.

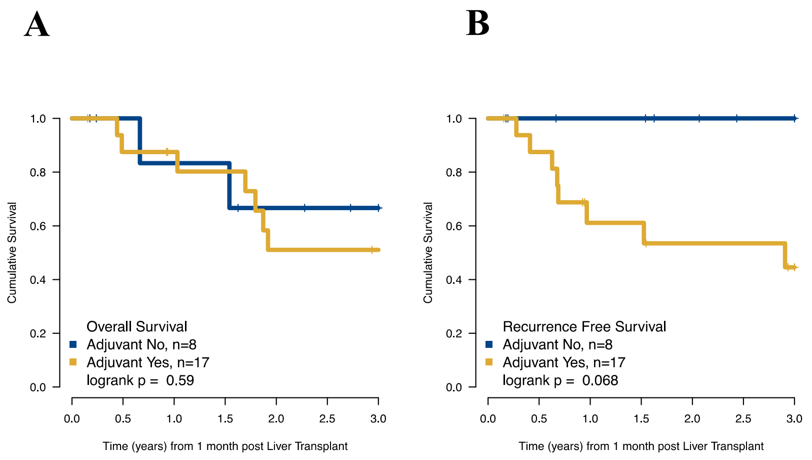

Supplement: Supplementary file 1 [file txd-11-e1760-s001.pdf]
